# Laboratory Calibration and Field Validation of Soil Water Content and Salinity Measurements Using the 5TE Sensor

**DOI:** 10.3390/s19235272

**Published:** 2019-11-29

**Authors:** Nessrine Zemni, Fethi Bouksila, Magnus Persson, Fairouz Slama, Ronny Berndtsson, Rachida Bouhlila

**Affiliations:** 1 National Institute for Research in Rural Engineering, Water, and Forestry, Box 10, Ariana 2080, Tunisia; bouksila.fethi@iresa.agrinet.tn; 2 Laboratory of Modelling in Hydraulics and Environment, National Engineering School of Tunis, University of Tunis El Manar (ENIT), Box 37, Le Belvédère Tunis 1002, Tunisia; fairouz.slama@enit.utm.tn (F.S.); rachida.bouhlila@enit.utm.tn (R.B.); 3 Department of Water Resources Engineering, Lund University, Box 118, SE-221 00 Lund, Sweden; magnus.persson@tvrl.lth.se (M.P.); ronny.berndtsson@tvrl.lth.se (R.B.); 4 Centre for Middle Eastern Studies, Lund University, Box 201, SE-221 00 Lund, Sweden

**Keywords:** soil salinity, soil water content, FDR sensor, soil pore water electrical conductivity, sensor calibration and validation, real time monitoring

## Abstract

Capacitance sensors are widely used in agriculture for irrigation and soil management purposes. However, their use under saline conditions is a major challenge, especially for sensors operating with low frequency. Their dielectric readings are often biased by high soil electrical conductivity. New calculation approaches for soil water content (*θ*) and pore water electrical conductivity (ECp), in which apparent soil electrical conductivity (ECa) is included, have been suggested in recent research. However, these methods have neither been tested with low-cost capacitance probes such as the 5TE (70 MHz, Decagon Devices, Pullman, WA, USA) nor for field conditions. Thus, it is important to determine the performance of these approaches and to test the application range using the 5TE sensor for irrigated soils. For this purpose, sandy soil was collected from the Jemna oasis in southern Tunisia and four 5TE sensors were installed in the field at four soil depths. Measurements of apparent dielectric permittivity (Ka), ECa, and soil temperature were taken under different electrical conductivity of soil moisture solutions. Results show that, under field conditions, 5TE accuracy for θ estimation increased when considering the ECa effect. Field calibrated models gave better θ estimation (root mean square error (RMSE) = 0.03 m^3^ m^−3^) as compared to laboratory experiments (RMSE = 0.06 m^3^ m^−3^). For ECp prediction, two corrections of the Hilhorst model were investigated. The first approach, which considers the ECa effect on K’ reading, failed to improve the Hilhorst model for ECp > 3 dS m^−1^ for both laboratory and field conditions. However, the second approach, which considers the effect of ECa on the soil parameter K_0_, increased the performance of the Hilhorst model and gave accurate measurements of ECp using the 5TE sensor for irrigated soil.

## 1. Introduction

In arid and semiarid countries, such as Tunisia, irrigation is necessary for improved agricultural production. Water resources with good quality are limited, resulting in the use of low-quality irrigation water. This can induce soil salinization, leading to crop yield reduction, decreasing the agricultural productivity, and causing general income loss [1,2]. Thus, accurate monitoring of soil salinity in time and space is of great importance for precision irrigation scheduling to save water and avoid soil degradation. Over the last decades, soil dielectric sensors have been developed to measure apparent electrical conductivity (ECa) from which real soil salinity, the soil pore electrical conductivity (ECp), can be estimated [3]. Time domain reflectometry (TDR) has been established as the most accurate dielectric technique to estimate both volumetric water content (θ) and ECp in soils providing automatic, simultaneous, and continuous readings [4]. The efficiency of the TDR method has led to development of other techniques based on similar principles, such as capacitance methods. Some examples are the WET (Delta-T Devices Ltd., Cambridge, UK) and the 5TE (Decagon Devices Inc., Pullman, WA, USA) sensors, both based on frequency domain reflectometry (FDR). Compared to TDR, FDR sensors use a fixed frequency wave instead of a broad-band signal that makes them cheaper and smaller [5]. Dielectric methods are based on determination of apparent soil electrical conductivity (ECa) and soil apparent dielectric permittivity (Ka) [6]. Many models for the relationships between Ka and θ [4,7], ECa-θ, and ECa-ECp-Ka have been proposed in recent research [3,8,9,10]. However, dielectric properties are affected by physical and chemical soil properties. For example, high ECa affects the wave propagation, leading to errors in the estimation of Ka [11,12]. Thus, it is important to improve θ and ECp prediction models. 

Hilhorst [8] presented a theoretical model describing a linear relationship between ECa and Ka to predict ECp. This linear model can be used in a wide range of soil types without soil-specific calibration. Persson [13] evaluated the Hilhorst model using TDR in three sandy soils and confirmed the accuracy of the linear model with significant dependency on soil type. Many researchers [14,15,16,17] have tested the Hilhorst model using the WET sensor and showed that it can be improved with soil specific calibration. Using the WET sensor, improved correction of the Hilhorst model was proposed by Bouksila et al. [18], using loamy sand soil with about 65% gypsum. They found that the accuracy of ECp prediction is very poor when using standard soil parameters (K_0_). Thus, they proposed a correction by introducing a third-order polynomial fitted to the K_0_–ECa relationship instead of using the default K_0_. Kargas et al. [6] introduced a linear permittivity corrected model, proposed by Robinson et al. [5], in the Hilhorst relationship. They found that the correction depends on soil characteristics and that it is valid for ECa close to 2 dS m^−1^. These approaches consider the ECa effect on the prediction of ECp. However, research has not been performed using simultaneous controlled laboratory and field-scale experiments where effects of heterogeneity, root density, insect burrowing, etc., affect the observations [19]. Ideally, sensor calibration should be performed in structured soils due to its importance for pore size distribution and associated matrix potential [20]. Research has shown that calibration in repacked soil columns differs from calibration in disturbed soil used in laboratory experiments [21]. In addition, intrinsic soil factors such as soil temperature, presence of gravel, and microorganisms affect the soil structure and porosity contributing to the variability in ECa and Ka measurements under field conditions as compared to measurements in the laboratory [19].

Nowadays, farmers are embracing precision agriculture using sensors with high accuracy and low cost to increase yields and maintain the sustainability of irrigated land. The 5TE dielectric soil sensor, which also uses the Hilhorst model for ECp estimation, was introduced in 2007 and it is much cheaper than the WET sensor [22]. Several recent studies have investigated the 5TE probe in agricultural applications [2,23,24]. The 5TE sensor has electrodes at the end of the probe that are influenced by soil density making them sensitive to any variation in soil structure and θ content [25]. Despite this fact, most studies on the 5TE sensor performance [16,26,27] have been carried out under laboratory conditions. Thus, almost no research has been done in the field for testing its performance for ECp estimation, neither with the most used linear Hilhorst model nor with the more recent ECp approach proposed in literature. Another important practical aspect is to determine the application range of these sensors for irrigated soils under saline conditions. For example, it is important to determine at what ECa threshold the dielectric losses are no longer negligible and need to be corrected for. Furthermore, there is a lack of understanding of how laboratory calibration can be translated into field conditions. Thus, the sensors must be calibrated and validated under both conditions in order to assess the errors associated with translating one to the other [28]. 

In view of the above, the objective of the present study was to assess the performance of the 5TE sensor to estimate soil water content and soil pore electrical conductivity for a representative sandy soil used for cultivation of date palms. Both standard models and a novel approach using corrected models to compensate for high electrical conductivity were used. Results from both field and laboratory experiments were compared. The location of the field experiments was the Jemna oasis, southern Tunisia.

## 2. Materials and Methods

Soil parameter acronyms, data source, sensor specification and models used in the present work were presented in Appendix A.

### 2.1. Theoretical Considerations 

Any porous medium, such as soils, can be characterized by its permittivity, which is a complex quantity (K) composed of a real part (K’) describing energy storage, and an imaginary part (K’’) describing energy loss:

(1) $$K=K^{\prime }-j\;K^{\prime \prime }\;\mathrm{with}\;j=\surd -1$$

For soils with low salinity, it is often assumed that the polarization and conductivity effects can be neglected [4]. Under such conditions, the effect of K” is eliminated and K’ becomes equal to K, represented by Ka as the apparent dielectric constant [4]. Under saline conditions, the imaginary part of the dielectric permittivity increases with ECa, leading to error in the permittivity measurement. This problem becomes important for frequencies lower than 200 MHz [6]. According to Campbell [29], for a frequency range of 1–50 MHz, conductivity is the most important mechanism related to energy loss. However, using the hydra impedance probe, Kelleners and Verma [30] found that, in general, the total energy loss is related to relaxation loss except for fine sandy soil, where it is equal to zero at 50 MHz.

#### 2.1.1. Permittivity-Corrected Linear Model 

Many researchers [5,17,31,32] have studied how well low-frequency capacitance sensors measure Ka and to what degree it is affected by K’’. In general, it has been shown that the most important factor to consider is the conductivity effect on Ka, whereas the effect of relaxation losses appears to be small [4,6]. Thus, it is possible to correct the Ka reading by introducing a term for the ECa effect. Based on the work of Whalley [32], Robinson et al. [5] proposed a permittivity-corrected linear model where the theoretical permittivity can be considered equivalent to the refractive index of measurements by the TDR. Robinson et al. [5] conducted experiments using TDR and capacitance dielectric sensor in sandy soils with high ECa levels (up to 2.5 dS m^−1^) and they proposed a linear model that includes the ECa effect on the Ka prediction according to:

(2) $$\sqrt{K^{\prime }}=\sqrt{\mathrm{Ka}}-0.628\;\mathrm{ECa}$$

From this equation, we notice that the increase of ECa (dS m^−1^) leads to an increase in Ka. Using Equation (2), a corrected permittivity K’ can be determined eliminating the ECa effect [6].

#### 2.1.2. Water Content Model

The dielectric constant is about 80 for water (at 20 °C), 2 to 5 for dry soil, and 1 for air. Therefore, Ka is highly dependent on θ. Various equations for the Ka vs. *θ* relationship have been published. The most used θ-model is a third-order polynomial [4]. However, Ledieu et al. [7] showed that there is a simpler linear relationship for the θ prediction with only two empirical parameters, of the form:

(3) $$\theta =a\sqrt{\mathrm{Ka}}+b$$

where a and b are fitting parameters.

**Figure 1:**
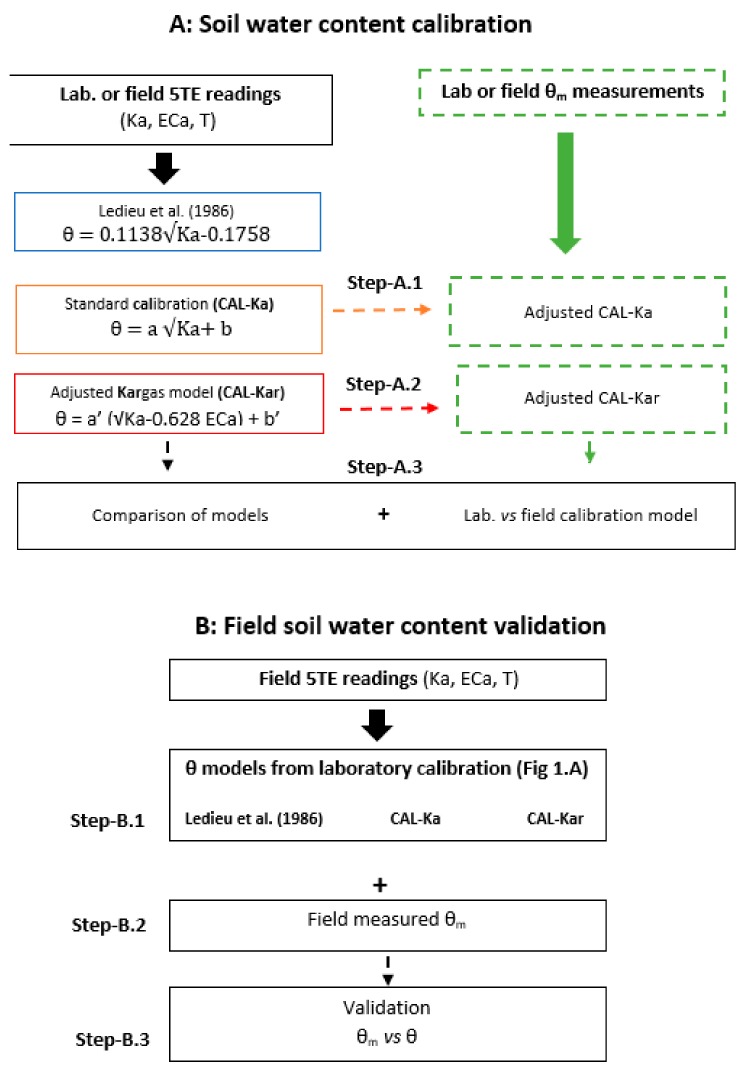
Schematic of θ calibration and validation possibilities investigated in the present study.

Figure 1 shows a schematic of calibration and validation possibilities for *θ* estimations that were used in the present study. The calibration consisted of fitting of parameters in different models (Figure 1). Optimal values for a and b, vs. a’ and b’ were determined by linear regression in the relationship √Ka-θ_m_ denoted as the CAL-Ka model (Figure 1, Step-A.1) and √K’-θ_m_ denoted as CAL-Kar model (Figure 1, Step-A.2), respectively. The θ_m_ was measured in experiments for different salinity levels. The standard Ledieu et al. [7] model (Figure 1) was used for comparison purposes as it is the simplest known model for mineral soil. The different steps (A.1 and A.2) were first completed using laboratory experiments (laboratory calibration) and then using field data (field calibration). The laboratory and field calibrated models were then compared with each other (Figure 1, Step-A.3). Finally, we used field data (step B.1, B.2, and B.3) to validate the laboratory experiments (laboratory model validation).

#### 2.1.3. Pore Water Electrical Conductivity Model

Different studies [33,34] have shown that ECa depends on both θ and ECp. Malicki et al. [35] and Malicki and Walczak [9] found that for Ka > 6 and when ECp is constant, the relationship between Ka and ECa is linear. An empirical ECp–ECa–Ka model has, thus, been proposed. Based on their results, Hilhorst [8] presented the following equation applicable when θ ≥ 0.10 m^3^ m^−3^:

(4) $$\mathrm{ECp}=\left(\frac{K_{w}\;}{\left(\mathrm{Ka}-K_{0}\right)}\;\right)\times \mathrm{ECa}$$

where K_w_ is the dielectric constant of the pore water (equal to 80.3) and K_0_ is a soil parameter equal to Ka when ECa = 0 (see [8], for details). According to Hilhorst [8], the K_0_ parameter depends on soil texture but is independent of ECa. He found the range of K_0_ to be between 1.9 and 7.6. For best results, this should be determined experimentally for each soil type. For most soils, a value of 4.1 has been recommended. One should notice, that in the Hilhorst model (Equation (4)), the Ka, K_w_, and K_0_ represent the real part of the dielectric constant only. From the linear relationship ECp = f (ECa), the slope that is inversely proportional to ECp and intercept K_0_ can be determined.

**Figure 2:**
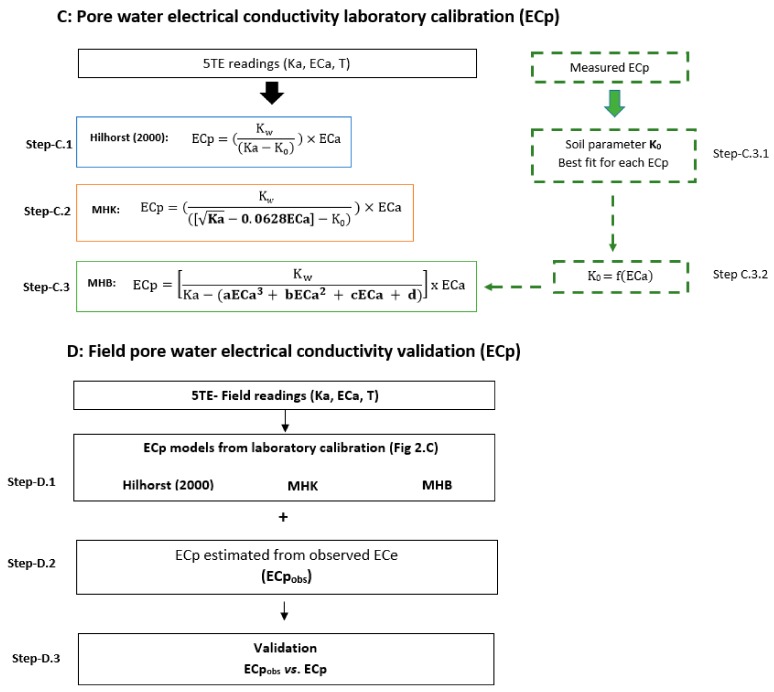
Schematic of electrical conductivity (ECp) calibration and validation used in the present paper.

In the present study, the Hilhorst model (Figure 2, Step-C.1) was tested using varying K_0_ soil parameters (4.1, 6 and 3.3). The K_0_ = 4.1 is the default value recommended by Hilhorst, K_0_ = 6 is the recommended value in the 5TE manual [36] while K_0_ = 3.3 is the value measured with distilled water according to the WET sensor manual [37].

Inspired by Bouksila et al. [18] and Kargas et al. [6], a modification of the Hilhorst model was investigated. Accordingly, a permittivity-corrected linear equation (Equation (2)) can be introduced in the Hilhorst model (Figure 2, Step-C.2) and ECp is predicted with two different K_0_ values (K_0_ = 4.1 and K_0_ = 3.3). Beside this, the soil fit parameter K_0_ is calculated for each salinity level by minimizing the mean square error (MSE) of the estimated ECp in the Hilhorst model (Step-C.3.1). The best fit K_0_ parameters are then plotted against ECa for the seven different ECp and a third-order polynomial function is determined (Step-C.3.2), and introduced in the Hilhorst model (Step-C.3). Finally, we used field data (step D.1, D.2, and D.3) to validate the laboratory experiments (laboratory model validation).

The temperature is an important factor influencing the electrical conductivity measurements; indeed, all ECa reading were adjusted in the present work using Equation (5). Besides, during experiments the temperature effect on K_w_ parameter was considered using the recommended temperature correction equation in the 5TE manual [36].

(5) $${\mathrm{ECa}}_{25}=\mathrm{ECa}\;\left[1-\left(\left(T-25\right)\;\times \;0.02\right)\right]$$

Measured Ka, ECa, and T in laboratory and field experiments are converted to ECp using the Hilhorst [8] model (Step-C.1), Kargas et al. [6] approach (Step-C.2), and Bouksila et al. [18] approach (Step-C.3), denoted as H, MHK, and MHB, respectively.

The different approaches in Figure 1 and Figure 2 have not been tested before using the 5TE sensor. The approaches CAL-Kar, MHK and MHB have previously only been tested once under controlled laboratory condition using the WET sensor. The novelty of the present work is to validate these approaches under field condition using the low cost capacitance sensor 5TE. In addition, the MHB approach developed by Bouksila et al. [18], used an experimentally determined K_0_ = f (ECa) relationship. Our new approach instead uses a K_0_ derived from best-fit parameter for each ECp level, which make the application of MHB approach much easier since there is no need for the K_0_ laboratory experiment.

Model performance for *θ* and ECp, was evaluated using both the root mean square error (RMSE) and coefficient of determination (R^2^). In addition, mean relative error (MRE) and coefficient of variation (CV) were used for ECp and *θ*, respectively. 

### 2.2. Study Area 

The field study was conducted in the Jemna oasis (33°36’15.”N, 9°00’39.”E), belonging to the Agricultural Extension and Training Agency (AVFA) located in the Kebeli Governorate, southern Tunisia. The oasis is equipped with a micro-irrigation system. The main crop is adult date-palm trees. The climate is arid with an annual rainfall of less than 100 mm, which is insufficient to sustain agriculture. The annual potential evapotranspiration is about 2000 mm [38]. Groundwater, situated at 17 m soil depth, with an electrical conductivity (ECiw) of about 3.5 dS m^−1^, is used for irrigation. The pH of groundwater is 7.8 and the geochemical facies is sodium chloride. Soil samples were collected from the top soil at 0–0.5 m depth. The soil was leached with distilled water in order to remove soluble salts and oven dried (105 °C) for 24 h. Then, the soil was passed through a 2 mm sieve. Soil particle size distribution was determined using the sedimentation method (pipette and hydrometer) and the electrical conductivity of saturated soil paste extract (ECe) was measured according to the United States Department of Agriculture (USDA) [39]. A summary of soil properties is presented in Table 1. 

### 2.3. Laboratory Experiments

Seven NaCl solutions with different electrical conductivity (0.02, 0.2, 0.5, 3.6, 5.3, 7.2, and 8.2 dS m^−1^) were prepared for the infiltration experiments. The soil was initially mixed with a small amount (about 0.05 m^3^ m^−3^) of the same water as used in the infiltration experiments to prevent water repellency. The soil was repacked into a plexiglas soil columns, 0.12 m in diameter and 0.15 m long (Soil Measurement System, Tucson, Arizona), to the average dry bulk density encountered in the field (about 1450 kg m^−3^). 

The 5TE sensor was used for observations [23]. It is a multifunctional sensor measuring Ka, ECa, and T (for more details, see Appendix A). The measuring frequency is 70 MHz and it is a three-rod type sensor with 0.052 m long prongs and 0.01 m spacing between adjacent prongs [23,40]. The 5TE probe was inserted vertically in the center of the column. Upward infiltration experiments were carried out by stepwise pumping a known volume of a NaCl solution (45 mL) with a precise syringe pump from the bottom of the column. Twenty minutes after each injection, three measurements of Ka, ECa, and temperature were taken and averaged. This procedure was repeated until saturation (0.40 m^3^ m^−3^) was reached. Four hours after reaching saturation, measurements were again taken and pore water was extracted from the bottom of the column with a manual vacuum pump. Electrical conductivity of extracted pore water ECp_m_ was measured with a conductivity meter. In total, seven upward infiltration experiments were conducted, one for every NaCl solution.

### 2.4. Field Measurements 

Four 5TE sensors were installed between date-palm trees at four soil depths (0.10, 0.15, 0.30, and 0.45 m). The 5TE probes were connected to a Decagon Em50 data logger. The DataTrac3 software version 3.15 [23] was used to download collected data from the Em50. Volumetric soil water content and pore electrical conductivity were estimated using standard parameters of the Ledieu et al. [7] and Hilhorst [8] models, respectively. In addition, soil samples were taken by hand auger at the same depth of sensor installation on 24 April and 3 October 2018. Gravimetric water content θ_m_ and electrical conductivity of saturated soil paste extract (ECe) were measured in laboratory according to USDA standards. The soil dry bulk density (Bd) was measured in the field using the cylinder method at five soil depths (0.1 m depth intervals to 0.5 m). During April 2018, the average soil Bd was equal to 1.43 g cm^−3^ and varied from 1.3 to 1.6 g cm^−3^. 

## 3. Results

### 3.1. Soil Water Content

**Figure 3:**
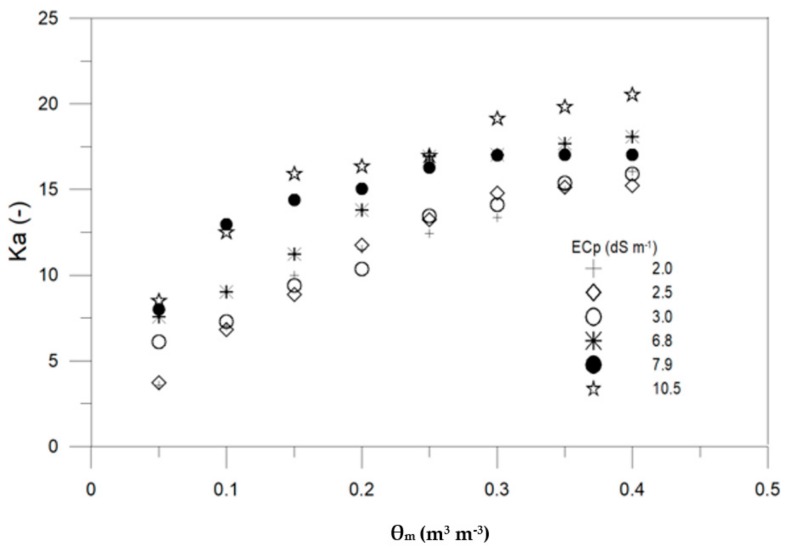
Apparent dielectric permittivity (Ka) vs. measured volumetric water content (θ_m_) for various pore electrical conductivity (ECp) levels (dS m^−1^).

Figure 3 presents the relationship between Ka and observed *θ_m_* with different salinity levels (ECp, dS m^−1^) measured during the upward infiltration experiments. For largest ECp, ECa did not exceed 2.5 dS m^−1^. It is seen that ECa considerably affects the Ka readings, especially for high ECp. This can lead to significant errors for both Ka and ECa, indicating that 5TE probe readings need to be corrected when used in saline soils. The overestimation of Ka as ECa increases has been described by several authors (e.g., [19,27]).

**Figure 4:**
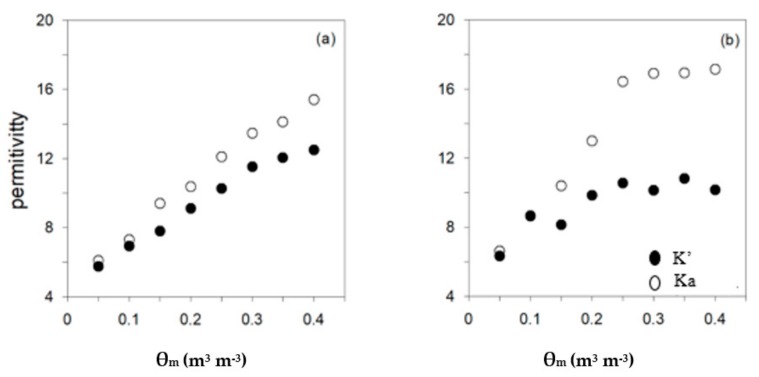
Relationship Ka-*θ_m_* (open circles) and K’-*θ_m_* (filled circles) using the 5TE sensor for ECp = 3 dS m^−1^ (**a**) and ECp = 9.8 dS m^−1^ (**b**).

In Figure 4, Ka and K’ (corrected with Equation (2)) for two ECp levels (3 and 9.8 dS m^−1^) are plotted against measured *θ_m_*. K’ values are very close to Ka when ECp ≤ 3 dS m^−1^, especially at low *θ* (θ ≤ 0.15 m^3^ m^−3^ and ECa ≤ 0.43 dS m^−1^). However, for ECp = 9.8 dS m^−1^, the difference between Ka and K’ is more pronounced, especially for θ ≥ 0.15 m^3^ m^−3^ and ECa ≥ 0.75 dS m^−1^.

The calibrated parameters using laboratory data for CAL-Ka and CAL-Kar approaches are presented in Table 2. For all models tested under laboratory conditions, RMSE increased with ECp. Soil water content from CAL-Kar approach matched well measured θ_m_ for ECp ≤ 3 dS m^−1^ (ECa < 0.7 dS m^−1^) and gave the best θ estimation compared to the Ledieu et al. [13] model and the soil-specific calibration CAL-Ka. However, for ECp ≥ 6.8 dS m^−1^, the CAL-Ka approach gave lower RMSE compared to the CAL-Kar model. For high ECp (≥ 6.8 dS m^−1^), the performance of the CAL-Kar model deteriorated.

### 3.2. Field Validation of Soil Water Content Models

During field experiments, Ka measured by the four 5TE probes varied from 6.5 to 11, ECa from 0.17 to 0.75 dS m^−1^, and measured soil moisture (*θ_m_*) from 0.10 to 0.24 m^3^ m^−3^. According to R^2^ of field validation results (Table 2), the best model to predict θ under field conditions is CAL-Kar followed by CAL-Ka. However, RMSE analysis indicates that there is no significant difference between observed and estimated θ using both approaches, implying that both predicted θ accurately for ECa ≤ 0.7 dS m^−1^.

**Figure 5:**
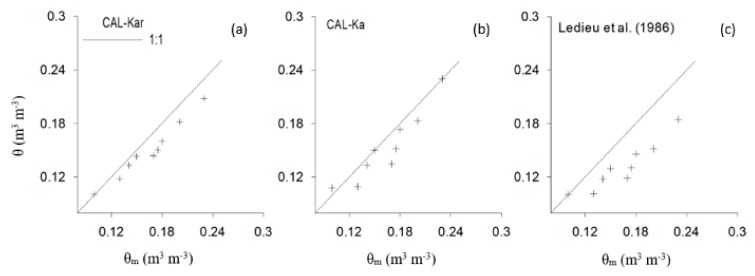
Estimated soil water content (*θ*) vs. measured (*θ*_m_) using CAL-Kar approach (**a**), CAL-Ka approach (**b**) and Ledieu et al. [13] model (**c**) under field conditions, solid line gives the 1:1 relationship.

From Figure 5, a slight underestimation of the different models is observed and this is more pronounced for the Ledieu et al. [7] model. The underestimation can be related to adsorbed water, resulting in a lower amount of mobile water in the soil, thus reducing the Ka readings (detection) by the 5TE sensor and eventually resulting in underestimation of Ka [41,42]. The difference between observed and predicted θ may also be attributed to variability in soil structure, bulk density, presence of stones, roots, and other inert material in the core samples. The difference may also be linked to the spatial variability of θ between sampled and monitored soils. Similar findings have been reported for mineral soils using the 5TE sensor [41], for Luvisol using the 5TM capacitance sensor [42], and using the ECH2O sensor in sandy soil [43]. The success of CAL-Ka and CAL-Kar models to calculate θ at field conditions is closely linked to the low range of ECa data measured by the 5TE sensor, below 0.7 dS m^−1^, during the period of investigation.

For the same range of soil salinity, RMSE was higher for the field as compared to laboratory data. For laboratory experiments, soil was crushed, washed, and passed through a 2 mm sieve. This means that its structure was changed as well as the pore size distribution, and some of the organic matter may have been removed. This allows more mobile water compared to field conditions [44]. As well, for field conditions, observed Bd profiles are not uniform and may vary with time. In contrast to the controlled laboratory experiments (e.g., constant Bd), the field Bd spatial and temporal variation will induce an additional error when laboratory models are used to estimate *θ*. 

We used the field data to calibrate the CAL-Ka and CAL-Kar models, the calibrated parameters for the models are presented in Table 2 (Field calibration). The RMSE decreased from 0.06 to 0.04 m^3^m^−3^ and from 0.06 to 0.03 m^3^ m^−3^ for CAL-Ka and CAL-Kar, respectively. Thus, the CAL-Kar approach gave better field predictions of θ. Similarly, Kinzli et al. [45] reported that field calibration was most successful for sandy soils. According to this finding, we may support the earlier conclusion that the permittivity corrected (CAL-Kar) model is recommended under field conditions if ECa is below 0.75 dS m^−1^. However, the Ledieu et al. [7] model cannot be used safely under field conditions in the case when soil specific calibration is not available. 

### 3.3. Soil Pore Electrical Conductivity (ECp)

#### 3.3.1. ECp Laboratory Calibration

Table 3 presents the RMSE for the different models. All models showed good performance in the 0–3 dS m^−1^ range, except Hilhorst with (K_0_ = 6) and MHK with K_0_ = 4.1. Moreover, RMSE results (Table 3), showed an increase of the range of default H model validity until ECp = 6.8 dS m^−1^. This finding can be linked to the higher operating frequency of 5TE (70 MHz) compared to the capacitance sensor used by Hilhorst (30 Mhz). Hilhorst reported that the model assumption ceases to be accurate at higher salinity as ECp significantly deviates from that of free water. 

From the results presented in Table 3, the ECp limit for accurate measurements seems to be 6.8 dS m^−1^. Similar results were reported by Scudiero et al. [40], using the 5TE sensor and ECp limit <10 dS m^−1^ with RMSE equal to 0.68 dS m^−1^. Using the H model with K_0_ value recommended in the Decagons manual (K_0_ = 6) showed a larger RMSE for all salinity levels compared the default parameter (K_0_ = 4.1). The H model with K_0_ = 3.3 (determined experimentally according to the WET manual) gave better results for the three salinity ranges. Persson [13] stated that the H model using a fitted soil parameter gives ECp values statistically similar to other model results (e.g., [3,10,46]).

Focusing on the modified Hilhorst model using the MHK approach with K_0_ = 4.1, one can observe that the RMSE is at maximum, especially for ECp ≥ 6.8 dS m^−1^. Kargas el al. [6] validated this approach using a lower salinity level (ECp ≤ 6 dS m^−1^). According to our results (Figure 7), an overestimation of the H model, especially at ECp ≥ 3 dS m^−1^, is observed. Similarly, Visconti et al. [19] showed an overestimation of ECp in the range of 0–10 dS m^−1^ and Scudiero et al. [40] showed an overestimation of ECp in the range 3–10 dS m^−1^, both working with the 5TE sensor and the H model. In the present study, the H model overestimated ECp, thus using the MHK approach will not improve results. 

The observed overestimation by the H model might be due to K_0_, which was assumed to be equal to 4.1. In addition, one should note that the H model does not consider solid particle surface conductivity, which could contribute to the ECp error [17]. From Table 3, decreasing K_0_ from 4.1 to 3.3 for both the H and MHK model leads to a significant decrease of RMSE, two times lower than the default. The H model seems to be more dependent on the soil parameter K_0_ than on Ka and ECa.

**Figure 6:**
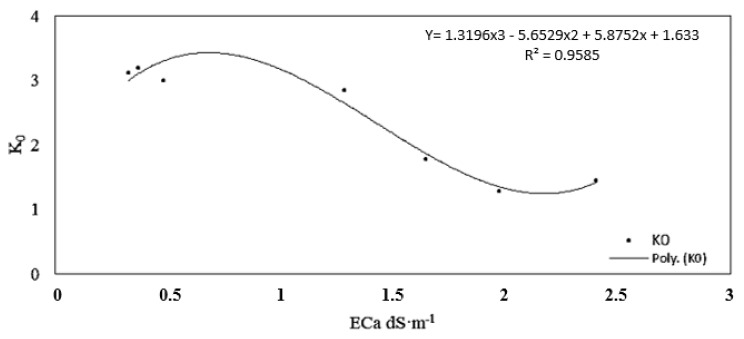
Best fit soil parameter (K_0_) vs. bulk soil electrical conductivity (ECa).

K_0_ estimated from the best fit approach for the different salinity levels is plotted against ECa in Figure 6. The K_0_ range varied between 1.29 and 3.2 with a mean of 3.0, which is similar to the K_0_ determined experimentally using distilled water (K_0_ = 3.3). 

At saturation, ECa was equal to 0.32 dS m^−1^ and 2.4 dS m^−1^ and Ka was equal to 15 and 19 for the lowest (2 dS m^−1^) and the highest (10.5 dS m^−1^) observed ECp, respectively. According to Figure 6, K_0_ decreases with increasing salinity. Similar to [18], our results showed that K_0_ is not constant, but depends on ECa and that a third-order polynomial fitted the K_0_–ECa relationship rather well (R^2^ ≥ 0.95). K_0_ = f (ECa) in Figure 6, was used in the H model to predict ECp. Compared to the H model, for the individual ECp levels, using the MHB model, RMSE decreased significantly.

**Figure 7:**
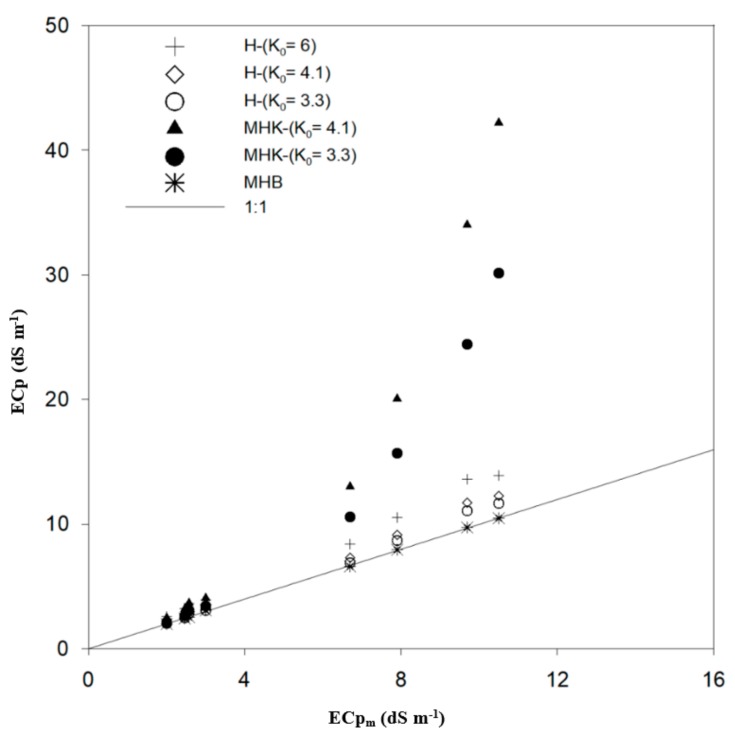
Estimated pore electrical conductivity (ECp) vs. measured for different model tested for laboratory conditions.

Figure 7 shows observed and predicted ECp using the H model with three different K_0_ and the MHK and MHB approaches, respectively. All model performances, are approximately the same for ECp ≤ 3 dS m^−1^, except when using K_0_ = 6 and K_0_ = 4.1 for H and MHK models, respectively. 

Based on the laboratory results, the MHB approach improved the H model and gave accurate estimation of ECp with R^2^ = 0.99 for all salinity levels. Thus, for high soil salinity (6.8 dS m^−1^ ≤ ECp ≤ 10.5 dS m^−1^), the MHB approach is recommended for achieving optimal accuracy of ECp measurements. For lower ECp (≤3 dS m^−1^), the standard H model is sufficient. For high ECp, the MHK approach failed to reproduce the observed ECp correctly and the approach is not recommended based on the results of our study. Further studies for different soil types are needed so that this combined approach in predicting ECp can be validated.

#### 3.3.2. Field Validation of ECp Models

Unfortunately, we do not have field observed ECp to validate and statistically compare the different models. Instead, we determined a linear relationship (ECp = f (ECe)) for different calculated ECp, using the H, MHK, and MHB models and 5TE measurements, with observed field ECe. Several researchers have studied relationships between ECe and ECp, e.g., [3], showing that the relationship is strongly linear. The relationship (ECp = f (ECe)) with the highest R^2^ = 0.9 was chosen to predict the field ECp values (ECp_obs_). During the investigation period, ECe was determined from soil samples, according to the USDA standard (collected at the same depth as the location of the 5TE sensors), ranging between 1.7 and 4.1 dS m^−1^. The relatively low soil salinity is due to a rainfall observed in the field one day before soil sampling. 

**Figure 8:**
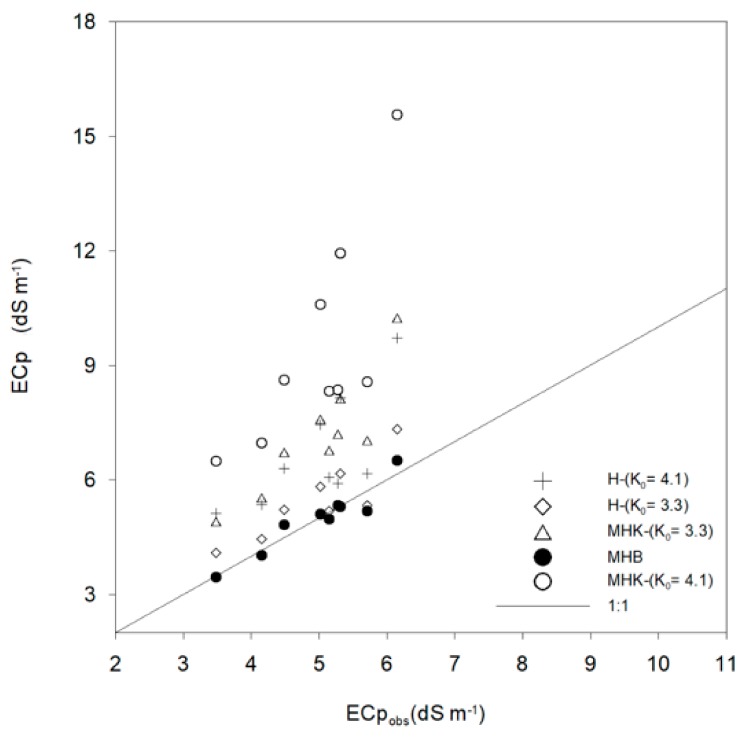
Estimated ECp vs. observed under field conditions.

The observed ECp_obs_ obtained from the best fit relationship is plotted against the estimated ECp for the different models in Figure 8. The H model with K_0_ = 6.6 was not included in the figure since it gave out of range values. The ECp estimation with MHB approach appears uniformly scattered about the 1:1 line. On the other hand, the H model with K_0_ = 3.3 shows a cloud of points near the 1:1 line. 

Compared to laboratory results, for the same ECa range (ECa ≤ 0.7 dS m^−1^) (Table 4), observed errors are higher for the field validation. The RMSE increased for all models. Errors are mainly related to a number of factors absent in the laboratory but present under field conditions. Due to this reason, a methodological approach composed by laboratory calibration and field validation is optimal. 

The MHB approach presents a significant improvement of the H model, especially at high ECp (Table 4). The H and MHK model fit is acceptable for field and laboratory conditions only for ECp ≤ 3dS m^−1^ while the MHB approach is acceptable for field conditions and it can be safely used for sandy soil and ECp ≤ 7 dS m^−1^.

Since variation and uncertainties in the field are higher, it is recommended to validate the calibrated models with field data. According to our results, the H model with K_0_ = 6 is not recommended either with laboratory nor field data. However, the reduction of K_0_ to 3.3 increased the performance of the model and it can be safely used for ECp < 3 dS m^−1^. For ECp > 3 dS m^−1^, the MHK approach did not improve the H model with RMSE more than 1 dS m^−1^ and it is not recommended. Thus, for achieving optimal accuracy of ECp measurements, the MHB approach is recommended for ECp ≤ 7dS m^−1^.

## 4. Conclusions 

In this study, the 5TE sensor performance for volumetric soil water content (*θ*) and soil pore electrical conductivity (ECp) estimation was investigated under laboratory and field conditions. First, two procedures for θ estimation based on a linear relationship of √Ka-*θ_m_* (CAL-Ka approach) and √K’-*θ_m_* (CAL-Kar approach) were investigated. Using the CAL-Kar approach, the effect of soil apparent electrical conductivity (ECa) on the real part of the complex dielectric permittivity (K’) was considered. In addition, the Ledieu et al. [7] relationship was used for comparison purposes. A site-specific validation of CAL-Ka and CAL-Kar models using 5TE field subset data and *θ* from soil samples at different depth was performed. Secondly, 5TE performance for soil salinity assessment was investigated using the H linear model according to correction proposed by Kargas et al. [6] (MHK model), and Bouksila et al. [17] (MHB model). The default value of soil parameter K_0_ = 4.1 and K_0_ = 6 recommended in the 5TE manual was used for comparison.

For soil water content, calibration considering the ECa effect on K’ increased the performance of the 5TE sensor under field conditions for ECa ≤ 0.75 dS m^−1^ (R^2^ = 0.97, RMSE = 0.06 m^3^ m^−3^). However, the error in predicting θ was highest (0.10 m^3^ m^−3^) when the Ledieu et al. [7] model was used. Indeed, this model cannot be safely used under field conditions. Thus, we conclude that field calibration of the 5TE sensor is recommended for accurate soil water content estimation. Soil pore electrical conductivity calibration results, show that the 5TE sensor limit using the default H model is equal to 6.8 dS m^−1^ with RMSE = 0.57 dS m^−1^ and MRE = 9%. The 5TE sensor manual value (K_0_ = 6) is not recommended. However, K_0_ = 3.3 increases model performance over the investigated salinity range. The MHK approach, introducing the permittivity correction in the H model, failed to reproduce the observed ECp correctly and it is not recommended. In the next step, considering the effect of ECa on the K_0_ soil parameter in the H model (MHB approach), it was found that the standard model improves and gives accurate estimation of ECp with R^2^ equal to 0.99 for all salinity levels. Under field conditions, the MHB approach gives the best results for sandy soils.

It is a challenge to perform real-time monitoring of irrigated land under high-saline conditions to provide sustainable agriculture and farmer income increase. Using θ and ECp observations, it was shown that a methodological approach composed of a laboratory calibration and field validation is necessary. Further studies, for different soil types, are needed to validate this combined approach in predicting ECp.

## Figures and Tables

**Table 1:** Particle size percentage, pH and electrical conductivity of saturated soil paste extract (ECe) of investigated soil samples.

Depth (m)	Clay (%)	Fine Silt (%)	Coarse Silt (%)	Fine Sand (%)	Coarse Sand (%)	pH	ECe (dS m^−1^)
0–0.5	5	3	4	22	65	8.5	1.8

**Table 2:** Root mean square error (RMSE, m^3^ m^3^), determination coefficient (R^2^) and coefficient of variation (CV,%) of estimated soil water content using Ledieu et al. [7], standard calibration (CAL-Ka) and permittivity corrected model (CAL-Kar) for different water pore electrical conductivity (ECp).

Laboratory Calibration				
**ECp (dS m^−1^)**		Ledieu et al. (1986)	CAL-Ka	CAL-Kar
	**Fit**	Equation (4)	θ = 0.16 √Ka^1^−0.30	θ = 0.18√K’^2^−0.33
**ECp ≤ 3**	RMSE	0.06	0.05	0.04
	R^2^	0.93	0.95	0.95
**ECp = 6.8**	RMSE	0.08	0.06	0.10
	R^2^	0.73	0.87	0.50
**6.8 < ECp ≤ 10.5**	RMSE	0.09	0.07	0.13
	R^2^	0.77	0.85	0.39
**Mean RMSE**		0.08	0.06	0.09
**Mean R^2^**		0.8	0.9	0.6
**CV (%)**		26.5	20	19.8
**Field calibration**				
	**Fit**		θ = 0.15 √Ka−0.26	θ = 0.20√K’−0.37
**ECa^3^ ≤ 0.7** **and** **1.7 ≤ ECe^4^ ≤ 4.1**	RMSE (m^3^ m^−3^)	-	0.04	0.03
	R^2^	-	0.94	0.97
	CV (%)	-	23	24
**Field validation**				
**ECa ≤ 0.7** **and** **1.7 ≤ ECe ≤ 4.1**	RMSE (m^3^ m^−3^)	0.1	0.060	0.060
	R^2^	0.80	0.88	0.97
	CV (%)	27	21	24

^1^ Apparent soil permittivity, ^2^ Corrected apparent soil permittivity, ^3^ Soil apparent electrical conductivity, ^4^ Electrical conductivity of saturated soil paste extract.

**Table 3:** Root mean square error (RMSE, dS m^−1^) of estimated pore electrical conductivity (ECp) using Hilhorst (K_0_ = 4.1, 3.3, and 6), modified Hilhorst according to Kargas et al. [6] (MHK) (K_0_= 4.1 and 3.3), and modified Hilhorst according to Bouksila et al. [18] (MHB) models.

ECp (dS m^−1^)	Hilhorst (2000)			MHK		MHB
Soil Parameter-K_0_	K_0_ = 4.1	K_0_ = 3.3 ^1^	K_0_ = 6	K_0_ = 4.1	K_0_ = 3.3 ^1^	Best Fit K_0_ = f (ECa^2^)
**ECp ≤ 3**	0.29	0.14	0.83	0.88	0.34	0.044
**ECp = 6.8**	0.57	0.21	1.7	6.3	3.8	0.050
**6.8 < ECp ≤ 10.5**	1.48	0.99	3.06	-	-	0.054

^1^ K_0_ soil parameter determined experimentally according to the method in the Wet sensor manual using distilled water. ^2^ Soil apparent electrical conductivity.

**Table 4:** Root mean square error (RMSE, dS m^-1^) and determination coefficient (R^2^) of Hilhorst (K_0_ = 4.1, 3.3, and 6), modified Hilhorst according to Kargas et al. [6] (MHK) (K_0_ = 4.1 and 3.3) and modified Hilhorst according to Bouksila et al. [18] (MHB) models field validation.

	Hilhorst (2000)		MHK		MHB	
ECa2 ≤ 0.7 and 1.7 ≤ ECe3 ≤ 4.1	K_0_ = 4.1	K_0_ = 3.3^1^	K_0_ = 6	K_0_ = 4.1	K_0_ = 3.3^1^	Best Fit K_0_ = f (ECa)
RMSE (dS m−1)	0.82	0.70	10	1.8	1.34	0.30
R^2^	0.53	0.73	0.26	0.56	0.77	0.90

^1.^ K_0_ soil parameter determined experimentally according to the method in the Wet sensor manual using distilled water. ^3^ Soil apparent electrical conductivity, ^4^ Electrical conductivity of saturated soil paste extract.

## Appendix A

**Table A1:** Soil parameter acronyms, data source, sensor specification and models used in the present work.

Soil Parameter	Acronym	Data Source	Sensor/Method
Soil dry bulk density	Bd	Measured	Cylinder method- United States Department of Agriculture (USDA)
Soil pH	pH	Measured	pH-meter
Apparent soil permittivity	Ka	Measured	5TE-probe
Soil parameter	K_0_	Estimated	5TE-probe
Dielectric constant of pore water	K_w_	Estimated	5TE-probe
Corrected apparent soil permittivity	K’	Estimated	5TE-probe
Soil temperature	T	Measured	5TE-probe
Electrical conductivity of saturated soil paste extract	ECe	Measured	EC-meter/USDA method
Soil apparent electrical conductivity	ECa	Measured	5TE-probe
Irrigation water electrical conductivity	ECiw	Measured	EC-meter
Measured soil water content	$\theta_{m}$	Measured	Gravimetric method-USDA
Estimated volumetric water content	θ	Estimated	θ –Models (see Figure 1)
Laboratory measured pore water electrical conductivity	ECp_m_	Measured	EC-meter
Field observed pore water electrical conductivity	ECp_obs_	Measured	ECp_obs_ = a ECe + b (see Figure 2)
Pore water electrical conductivity	ECp	Estimated	ECp-Models (see Figure 2)
5TE sensor specification			
Type	Specifics		
Sensor type	FDR (Frequency Domain Reflectometry)		
Power supply	+3.6 to +15 V		
Frequency	70 MHz		
Size	Length 10.9 cm (4.3 in) Width 3.4 cm (1.3 in) Height 1.0 cm (0.4 in)		
Measurement volume	300 cm^3^		
Direct output data	Ka, ECa, and T		
Indirect output data	θ and ECp		
Range (Ka, ECa)	1–80, 0–7 dS m^−1^		
Resolution (Ka, ECa)	0.1, 0.01 dS m^−1^		
Accuracy (Ka, ECa)	±3%, ±10%		
Models			
CAL-Ka (see Figure 1)	Calibration of soil water content model without permittivity correction		
CAL-Kar (see Figure 1)	Calibration of soil water content model with permittivity correction according to Kargas et al. (2017)		
H (see Figure 2)	Standard Hilhorst (2000) model for ECp prediction		
MHK (see Figure 2)	Modified Hilhorst model according to Kargas et al. (2017) for ECp prediction		
MHB (see Figure 2)	Modified Hilhorst model according to Bouksila et al. (2008) for ECp prediction		
Model performance statistic tool			
RMSE	Root Mean Square Error		
R^2^	Coefficient of determination		
MRE	Mean Relative Error		
CV	Coefficient of Variation		
